# Carcinoembryonic antigen potentiates non-small cell lung cancer progression *via* PKA-PGC-1ɑ axis

**DOI:** 10.1186/s43556-024-00181-3

**Published:** 2024-05-24

**Authors:** Juan Lei, Lei Wu, Nan Zhang, Xudong Liu, Jiangang Zhang, Liwen Kuang, Jiongming Chen, Yijiao Chen, Dairong Li, Yongsheng Li

**Affiliations:** 1 Department of Medical Oncology, Chongqing University Cancer Hospital, Chongqing, 400030 China; 2 School of Medicine, Chongqing University, Chongqing, 400030 China

**Keywords:** Carcinoembryonic antigen (CEA), Non-small cell lung cancer, Fatty acid metabolism, PGC-1α, Anti-tumor therapy

## Abstract

Carcinoembryonic antigen (CEA) is a tumor-associated antigen primarily produced by tumor cells. It has been implicated in various biological processes such as cell adhesion, proliferation, differentiation, and metastasis. Despite this, the precise molecular mechanisms through which CEA enhances tumor cell proliferation remain largely unclear. Our study demonstrates that CEA enhances the proliferation and migration of non-small cell lung cancer (NSCLC) while also inhibiting cisplatin-induced apoptosis in NSCLC cells. Treatment with CEA led to an increase in mitochondrial numbers and accumulation of lipid droplets in A549 and H1299 cells. Additionally, our findings indicate that CEA plays a role in regulating the fatty acid metabolism of NSCLC cells. Inhibiting fatty acid metabolism significantly reduced the CEA-mediated proliferation and migration of NSCLC cells. CEA influences fatty acid metabolism and the proliferation of NSCLC cells by activating the PGC-1α signaling pathway. This regulatory mechanism involves CEA increasing intracellular cAMP levels, which in turn activates PKA and upregulates PGC-1α. In NSCLC, inhibiting the PKA-PGC-1α signaling pathway reduces both fatty acid metabolism and the proliferation and migration induced by CEA, both in vitro and in vivo. These results suggest that CEA contributes to the promotion of proliferation and migration by modulating fatty acid metabolism. Targeting CEA or the PKA-PGC-1ɑ signaling pathway may offer a promising therapeutic approach for treating NSCLC.

**Supplementary Information:**

The online version contains supplementary material available at 10.1186/s43556-024-00181-3.

## Introduction

Lung cancer is a major contributor to cancer-related deaths globally, with non-small cell lung cancer (NSCLC) making up about 85% of cases [1]. Unfortunately, many patients are diagnosed with advanced-stage lung cancer, limiting their treatment options to palliative chemotherapy and resulting in a life expectancy of 1 to 2 years for most individuals [2]. Improving treatment outcomes and extending survival in advanced NSCLC patients is crucial, highlighting the need for innovative treatment approaches.

Early detection of lung cancer is widely recognized as a key factor in reducing mortality rates and increasing survival. Common diagnostic methods include imaging tests like chest X-ray, CT scan, and PET-CT [3], as well as the use of tumor markers produced by tumor cells or the body in response to tumor development [4]. Carcinoembryonic antigen (CEA) is a well-established tumor marker that has been studied for over 30 years [5]. Elevated CEA levels in the serum of lung cancer patients compared to healthy individuals make it a valuable diagnostic tool [6]. The concentration of CEA in the serum of lung cancer patients is significantly higher than that in healthy individuals, making it a valuable diagnostic marker [7]. Increased CEA expression has been linked to poor prognosis in NSCLC patients [8].

CEA, a member of the glycosylphosphatidylinositol (GPI)-linked immunoglobulin (Ig) superfamily, is characterized by its extracellular domain anchoring to the cell membrane *via* GPI anchors [9]. Besides being utilized as a tumor marker, recent research indicates that CEA plays various biological roles. These functions include involvement in intercellular adhesion, transmission of regulatory signals, control of cell proliferation, differentiation, apoptosis, angiogenesis, and immune response [10]. Nevertheless, the precise biological function of CEA in the progression of NSCLC is not yet fully comprehended.

As our comprehension of tumor biology advances, we are also uncovering the complex nature of tumor metabolism. Metabolic reprogramming is a critical feature of tumor cells [11]. While the modification of glycolytic metabolism, known as the Warburg effect, is regarded as a crucial aspect of tumor metabolism, tumor cells must also effectively manage energy production and biosynthetic pathways to support cell proliferation [12]. Recent studies have emphasized the importance of lipid metabolism, specifically increased de novo lipogenesis, as a novel hallmark of many aggressive cancers [13]. However, the molecular mechanisms behind the reprogramming of lipid metabolism in NSCLC remain unclear.

This study aims to explore the impact of CEA on proliferation and migration in NSCLC and elucidate the underlying mechanisms involved in regulating tumor metabolism, highlighting its potential as a therapeutic target for NSCLC treatment.

## Results

### CEA promotes proliferation, migration and invasion of NSCLC

**Fig. 1:**
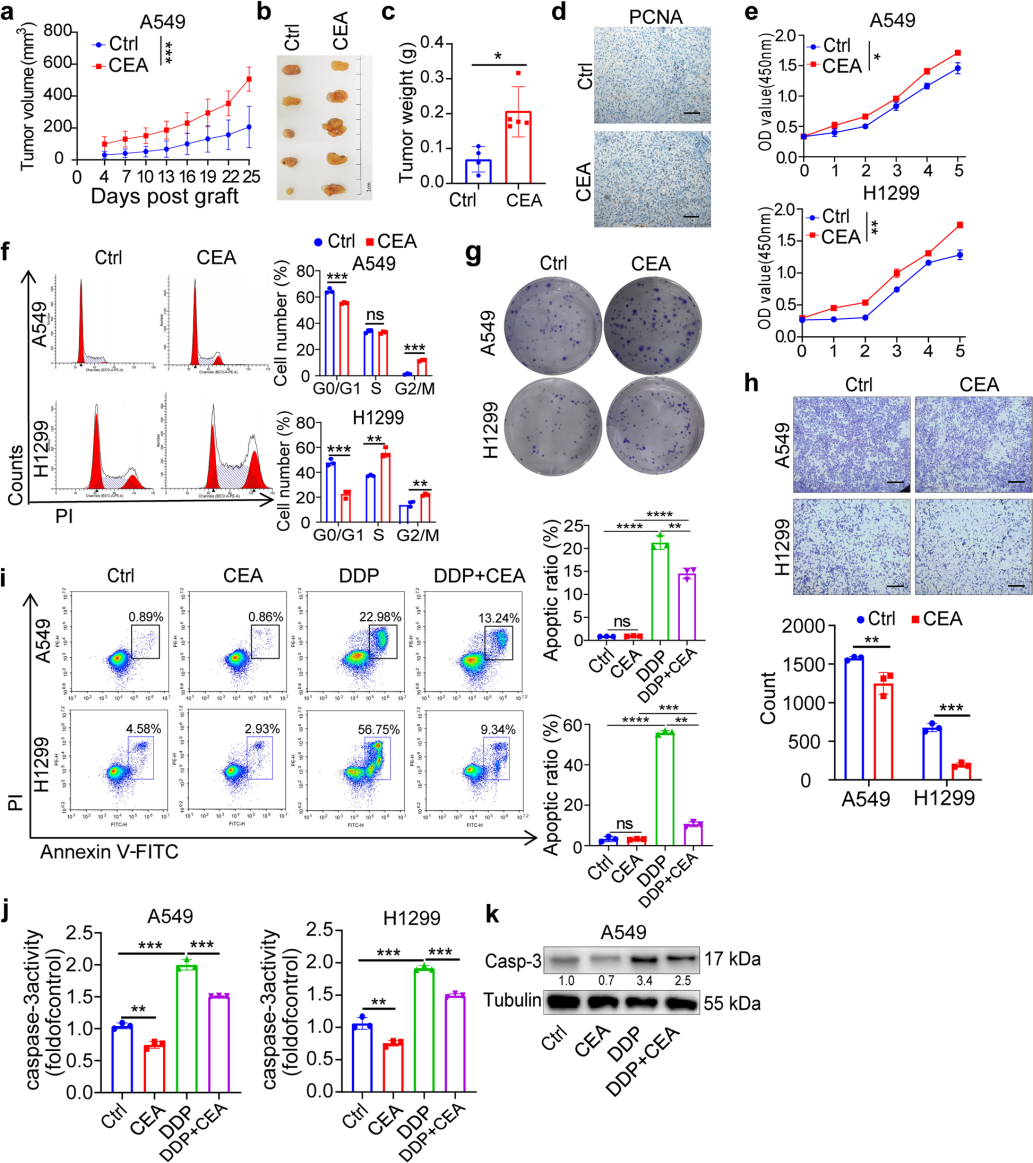
CEA promotes NSCLC growth in vivo and cell proliferation in vitro. **a**-**c** Nude mice were bearing A549 xenografts and treated with CEA (*i.p.* 50 μg/kg) for 25 days. growth curves were plotted (**a**), tumor volume (**b**), tumor weight (**c**) of A549 xenografts were shown,* n* = 5. **d** Representative immunohistochemistry of PCNA staining in xenografted tumors. Scale bar, 100 μm. e, f A549 and H1299 cells were treated with CEA (50 ng/mL) for 24 h. Cell viability was determined using the CCK-8 assay (**e**), Cell cycle of A549 and H1299 cells were determined (**f**). **g**, **h** Colony formation assays of A549 and H1299 cells (**g**), the transwell assays of A549 and H1299 cells after CEA treatment (**h**). **i**-**k** A549 and H1299 cells were exposed to CEA (50 ng/mL), DDP or combined for 24 h, the frequency of Annexin-V^+^ PI^+^ cells were determined by FCM (**i**), caspase-3 activity (**j**) and expression (**k**) were detected. Data were represented as mean ± SD, *n* = 3. *P < 0.05, **P < 0.01, ***P < 0.001, ****P < 0.0001, Two-tailed Student’s *t*-tests were used, *ns,* no significant difference

To assess the oncogenic impact of CEA in NSCLC in vivo, we conducted subcutaneous injections of A549 NSCLC cells into nude mice. Following 25 days of CEA treatment, the mice were euthanized, and the tumors were excised. Our results demonstrated a significant enhancement in the growth of A549 xenografts in nude mice upon CEA treatment (Fig. 1a). Furthermore, both the volume and weight of A549 tumors exhibited noticeable increases (Fig. 1b, c). Proliferating Cell Nuclear Antigen (PCNA) staining revealed a marked rise in A549 cell proliferation following CEA treatment (Fig. 1d). CCK8 assays confirmed the promotion of growth in both A549 and H1299 cells upon CEA treatment (Fig. 1e). Additionally, cell cycle analysis indicated a significant increase in the proliferation of A549 and H1299 cells with CEA treatment (Fig. 1f). Colony-formation assays displayed an enhanced clonogenic capacity of both A549 and H1299 cells with CEA treatment (Fig. 1g). Moreover, cell migration and invasion assays demonstrated that CEA treatment augmented the migration and invasion abilities of both A549 and H1299 cells (Fig. 1h). Cisplatin (DDP) is an anti-cancer agent known to induce tumor cell apoptosis through multiple mechanisms [14]. We further explored the potential of CEA to counteract DDP-induced apoptosis in A549 and H1299 cells. DDP treatment led to an increase in apoptotic cells in both A549 and H1299 cells (Fig. 1i). The activity and expression of the pro-apoptotic protein caspase-3 were elevated post-DDP administration (Fig. 1j, k). Conversely, CEA treatment reduced the number of apoptotic cells induced by DDP and decreased the activation of caspase-3, indicating an inhibitory effect of CEA on DDP-induced apoptosis. These findings suggest that CEA expression may play a role in the development of cisplatin resistance, underscoring the potential involvement of CEA in NSCLC proliferation, migration, invasion, and drug resistance.

### CEA accelerates NSCLC cells growth by reprogramming fatty acid metabolism

**Fig. 2:**
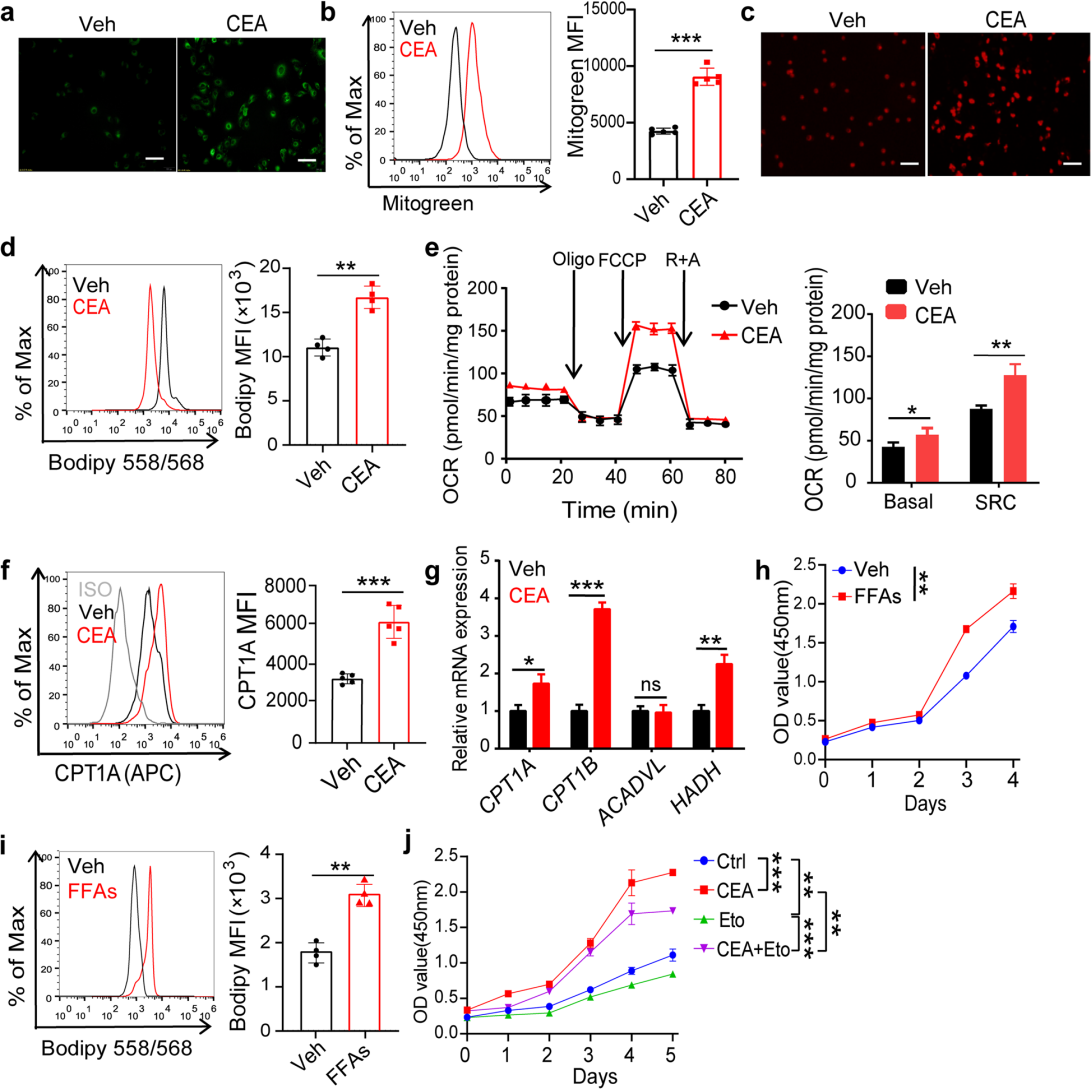
CEA reprograms lipid metabolism in NSCLC cells. **a**, **b** A549 cells were treated with CEA (50 ng/mL) for 24 h, Representative images (**a**) and quantification of mitogreen staining (**b**); *n* = 5. Scale bars: 20 μm. **c**, **d** Representative images (**c**) and quantification (**d**) of Bodipy 558/568 staining of A549 cells after CEA treatment for 24 h; *n* = 5. Scale bars: 20 μm. e A549 cells were treated with CEA for 24 h, oxygen consumption rates (OCR) and spare respiratory capacity (SRC) of veh (0.1% DMSO) and CEA treated A549 cells were measured using a Seahorse XFe 96 analyzer; *n* = 4. **f**, **g** A549 cells were treated with CEA for 24 h, CPT1A expression was measured by FCM (**f**); *n* = 4, and relative expression of *CPT1A*, *CPT1B*, *ACADVL* and *HADH* was determined by qPCR (**g**), *n* = 3. **h**, **i** A549 cells were treated with FFAs (0.4 mM) for 5 days, cell viability was determined (**h**), and quantification of Bodipy 558/568 staining of A549 cells after CEA treatment for 24 h (**i**); *n* = 4. **j** A549 cells were treated with CEA, Eto (200 µM) or combined for 5 days, cell viability was determined; *n* = 4. Data are expressed as means ± SD. *P < 0.05, **P < 0.01, ***P < 0.0001, Significance was assessed by Student’s *t* test

Fatty acid metabolism is a key player in the proliferation, migration, and invasion of NSCLC within the tumor microenvironment [14, 15]. Therefore, we hypothesized that CEA plays a significant role in enhancing the proliferation and migration of NSCLC cells by modulating fatty acid metabolism. Treatment with CEA resulted in an increase in mitochondria numbers and accumulation of lipid droplets in A549 and H1299 cells (Fig. 2a-d and Fig. S1a-d). Specifically, CEA-treated A549 cells showed higher mitochondrial oxygen consumption rate (OCR) and spare respiratory capacity (SRC), indicating enhanced fatty acid oxidation (FAO) (Fig. 2e). Moreover, there was an increase in fatty acid intermediates in both A549 and H1299 cells post-CEA treatment, suggesting an upregulation of fatty acid metabolism in the CEA-treated group (Fig. S2a). Consistent with this, the expression of carnitine palmitoyl transferase 1 (CPT1) and other key genes involved in FAO, such as *CPT1A*, *CPT1B*, *ACADVL* and *HADH*, were elevated in CEA-treated A549 cells (Fig. 2f, g). Further experiments illustrated that the altered fatty acid metabolism was responsible for the proliferative effects of CEA on A549 cells, as treatment with free fatty acids (FFAs) significantly boosted cell growth and intracellular lipid droplet accumulation [16] (Fig. 2h, i). Furthermore, inhibition of CPT1 with etomoxir (Eto) suppressed fatty acid metabolism genes and the proliferation of A549 cells (Fig. 2j). These results indicate that CEA drives the proliferation and metastasis of NSCLC cells by modulating fatty acid metabolism.

### CEA enhances PGC-1α activation to regulate fatty acid metabolism

**Fig. 3:**
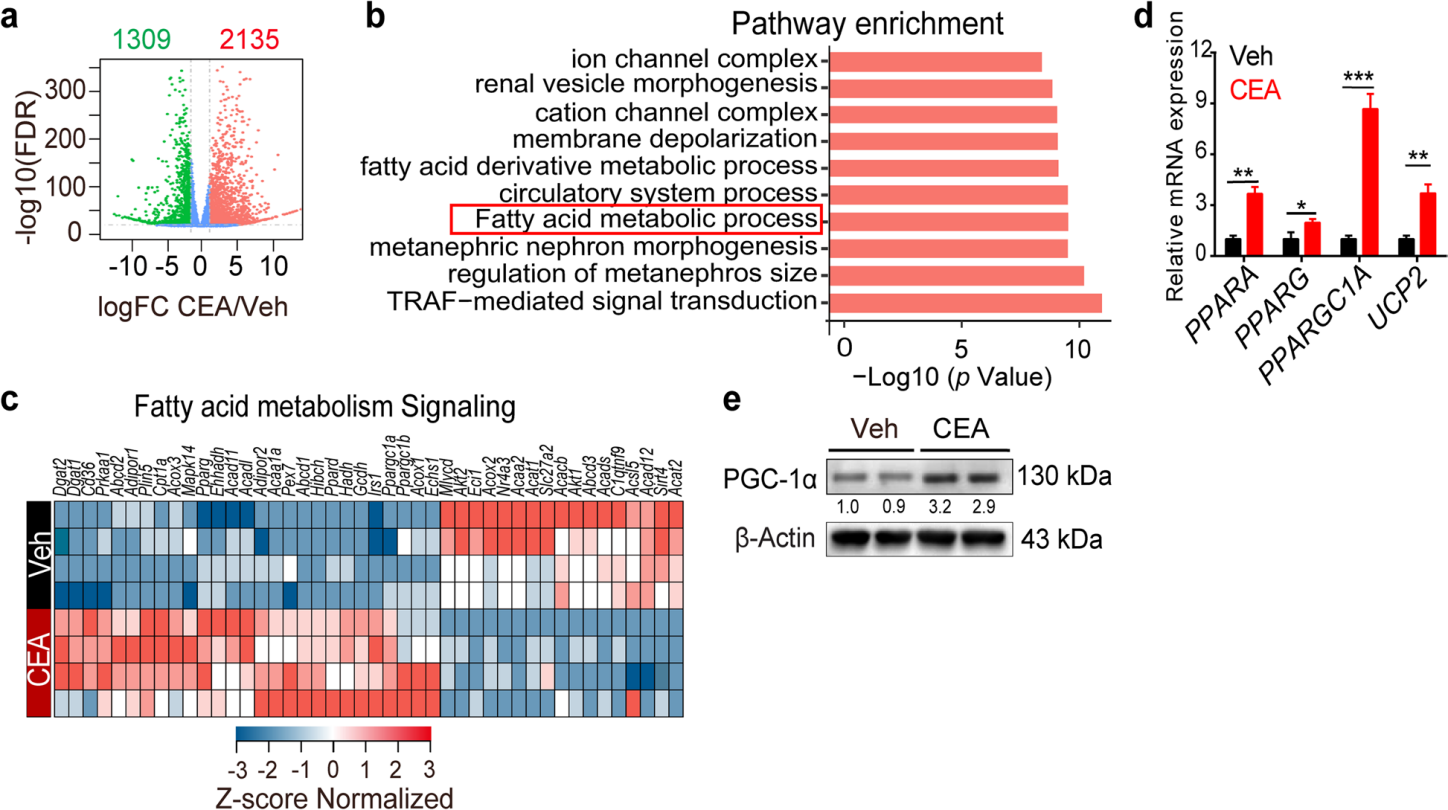
CEA enhances activated PGC-1α to regulate fatty acid metabolism. **a**-**c** Volcano plot (**a**), GO enrichment analysis (**b**) of the differentially expressed genes in Veh and CEA treated A549 cells, Heatmap of differentially expressed genes that involved in fatty acid metabolism (**c**), *n* = 3. **d** A549 cells were treated with CEA (50 ng/mL) for 24 h, *PPARA, PPARG, PPARGC1A* and *UCP2* expression were assessed; *n* = 3. **e** A549 cells were treated with CEA for 24 h, PGC-1α expression was determined by western blot analysis. Data are expressed as means ± SD. *P < 0.05, **P < 0.01, ***P < 0.0001, by two-sided unpaired student’s *t* test

To investigate the molecular mechanisms underlying the impact of CEA on fatty acid metabolism, we conducted RNA sequencing (RNA-seq) analysis on A549 cells treated with CEA in comparison to control cells. Our analysis revealed that CEA treatment led to differential expression of 1309 downregulated and 2135 upregulated genes in A549 cells as opposed to control cells (Fig. 3a). These genes were found to be enriched in various pathways, such as TRAF-mediated signal transduction, fatty acid metabolism, regulation of metanephros size, and metal ion transmembrane transporter activity (Fig. 3b). Importantly, our analysis indicated significant upregulation or downregulation of genes involved in fatty acid metabolism post-CEA treatment (Fig. 3c). These findings support the idea that CEA is involved in regulating the functions of fatty acid metabolism in A549 cells.

Peroxisome proliferator-activated receptors (PPARs) are a group of nuclear receptor proteins that play a key role in regulating fatty acid metabolism [17]. Upon treatment with CEA, A549 cells showed increased expression of PPARα, PPARγ, and PPAR-related genes (Fig. 3c, d), indicating sustained activation of the PPAR pathway. PPAR-γ coactivator (PGC)-1α is a transcription coactivator important for mitochondrial biogenesis and fatty acid metabolism [18]. Given the observed increase in mitochondria and impact on lipid metabolism following CEA treatment, we investigated the effect of CEA on PGC-1α expression in NSCLC cells. Our results revealed up-regulation of PGC-1α expression after CEA treatment in A549 cells (Fig. 3d, e), suggesting that CEA promotes fatty acid metabolism by activating PGC-1α.

### CEA promotes the proliferation and metastasis of NSCLC cells by activating PGC-1α

**Fig. 4:**
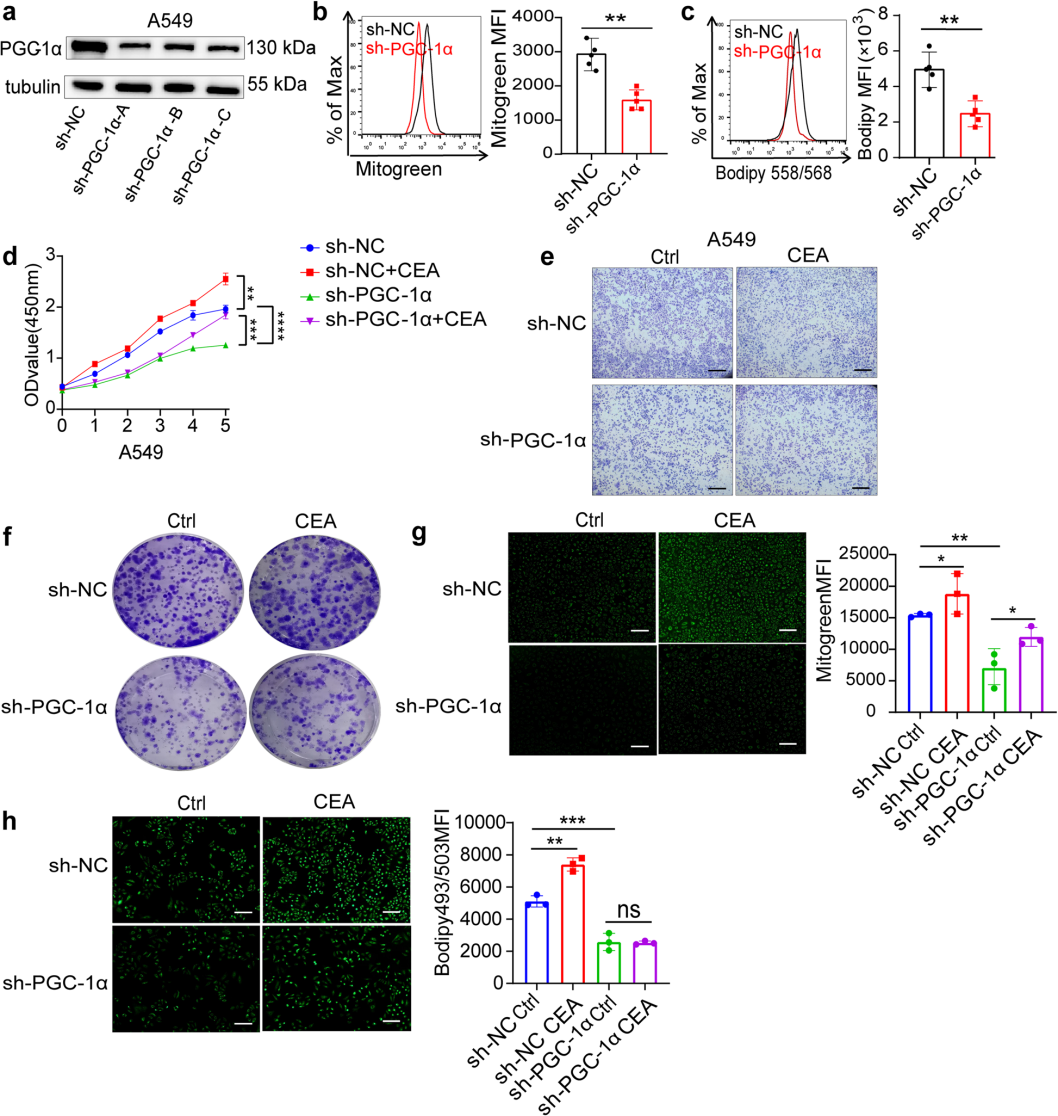
Knockdown of *PPARGC1A* attenuates CEA-mediated NSCLC cell proliferation in vitro. **a** Western blot analysis for PGC1-α expression in A549 cells after infected with three independent shRNAs targeting *PPARGC1A*. **b**, **c** Mitogreen staining (**b**) and Bodipy 558/568 staining (**c**) of A549 cells after CEA treatment for 24 h. *n* = 5. **d** CCK8 assays were performed to determine cell growth after *PPARGC1A* was knocked down and CEA treatment in A549 cells. **e** The abilities of migration and invasion of A549 cells after *PPARGC1A* knockdown. **f** Colony formation assays of A549 cells after *PPARGC1A* knockdown. **g**, **h** Mitogreen staining (**g**) and Bodipy 493/503 staining (**h**) of A549 cells after *PPARGC1A* was knocked down and CEA treatment. Scale bar, 100 μm. Data are expressed as means ± SD. *P < 0.05, **P < 0.01, ***P < 0.001, ****P < 0.0001, *ns,* no significant difference, by two-sided unpaired student’s *t* test

To examine the impact of PGC-1α on fatty acid metabolism and cell proliferation in NSCLC cells, three shRNAs were used to silence PGC-1α in A549 cells (Fig. 4a). This silencing resulted in reduced mitochondria numbers and increased lipid droplets accumulation (Fig. 4b, c). The downregulation of PGC-1α suppressed the expression of fatty acid metabolism-related genes like *CPT1A*, *CPT1B*, *ACADVL* and *HADH* induced by CEA (Fig. S3). Furthermore, the decrease in mitochondria numbers and lipid droplets accumulation was observed upon PGC-1α knockdown (Fig. 4b, c). Notably, the growth of A549 cells was significantly inhibited after PGC-1α knockdown, as shown by CCK8 assays (Fig. 4d). Silencing PGC-1α also reduced the invasion capabilities of A549 cells (Fig. 4e) and suppressed clonogenic capacity in colony-formation assays (Fig. 4f). The deficiency of PGC-1α hindered CEA’s ability to enhance mitochondria numbers and lipid droplets accumulation (Fig. 4g, h). These results suggest that CEA promotes the proliferation and metastasis of NSCLC cells by activating PGC-1α signaling.

### CEA upregulates PGC-1α expression *via* activating PKA signaling

**Fig. 5:**
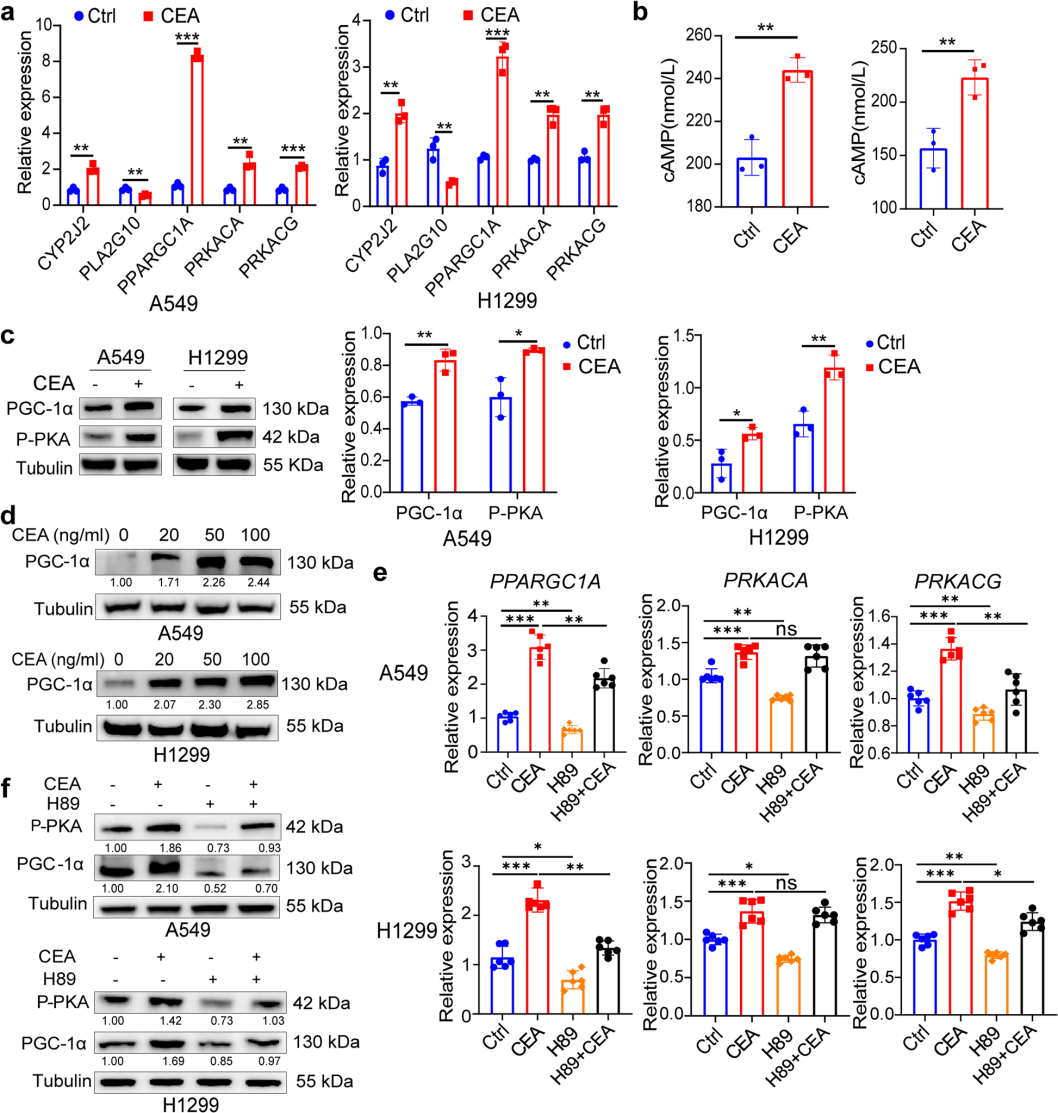
CEA enhances PGC-1α expression *via* PKA signal activation. **a** The gene expression of *PPARGC1A*, *PRKACA* and *PRKACG* in A549 and H1299 cells after CEA (50 ng/mL) treatment for 24 h were assessed; *n* = 3. **b** A549 and H1299 cells were treated with CEA for 24 h, the cAMP expression levels in cells were measured using an ELISA kit, *n* = 4. **c** A549 and H1299 cells were treated with CEA (50 ng/mL), PGC1-α and p-PKA expression were determined. **d** A549 and H1299 cells were treated with indicated concentration of CEA, PGC1-α protein expression were measured. **e**, **f** A549 and H1299 cells were treated with H89 in the presence or absence of CEA (50 ng/mL) for 24 h, relative expression of PGC1-α, PRKACA and PRKACG mRNA expression were determined by qPCR (**e**) *n* = 3, protein expression of PGC1-α and p-PKA were determined by Western blot analysis (**f**). Data are expressed as means ± SD. *P < 0.05, **P < 0.01, ***P < 0.001, *ns,* no significant difference, by two-sided unpaired student’s *t* test

Previous research has shown that activation of the cAMP and calcium signaling pathways leads to PGC-1α induction and mitochondrial biogenesis in muscle cells [19, 20]. Therefore, the study aimed to determine if CEA could elevate intracellular cAMP levels in NSCLC cells. The results revealed an upregulation of genes encoding cAMP-dependent catalytic α and γ protein subunits in lung cancer cells treated with CEA (Fig. 5a), indicating a potential increase in intracellular cAMP concentration and subsequent activation of PKA. Furthermore, it was observed that CEA treatment elevated intracellular cAMP levels in A549 and H1299 cells (Fig. 5b), in increased levels of p-PKA and PGC-1α (Fig. 5c, d). Inhibition of PKA with H89 led to a down-regulation of p-PKA and PGC-1α protein expression in these cells (Fig. 5e, f). Interestingly, H89 treatment suppressed CEA-induced gene expression of *PRKACG* but not *PRKACA* in A549 cells, and also inhibited fatty acid metabolism-related genes induced by CEA (Fig. S4a, b). These findings suggest that CEA can activate PGC-1α expression through the cAMP-PKA signaling pathway.

### Blocking PKA-PGC-1α axis blunts CEA-mediated NSCLC growth in vivo

**Fig. 6:**
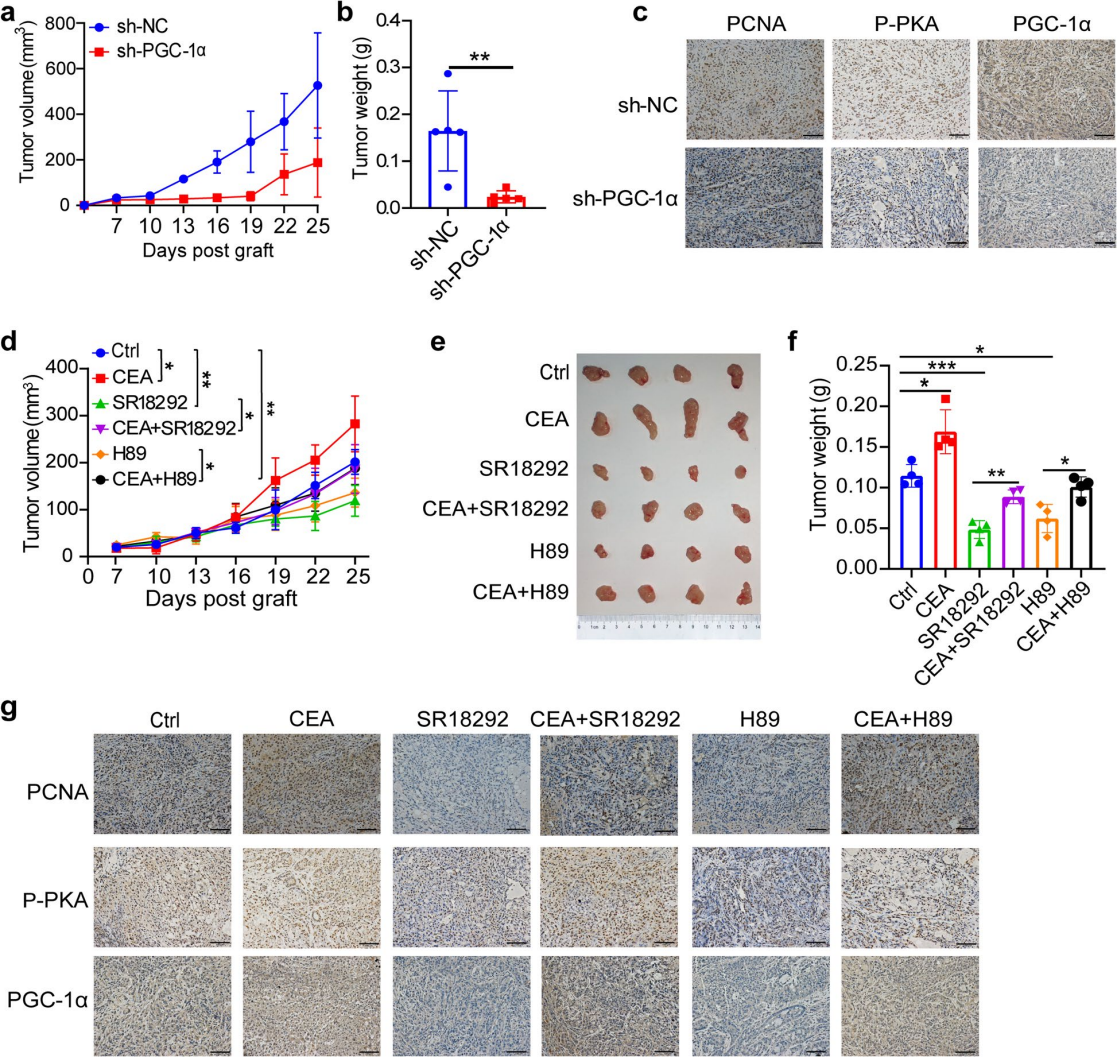
Blocking PKA-PGC-1α axis blunts CEA-mediated NSCLC growth in vivo. **a**, **b** sh-NC or PGC-1α knockdown A549 cells were subcutaneously injected into nude mice, tumor size was measured every 3 days (**a**), tumors xenografts were dissected at 25 days after transplantation, the tumor weight (**b**) was measured; *n* = 5. **c** Representative immunohistochemical images of PCNA, P-PKA and PGC-1α expression in A549 tumor tissues after PGC-1α knockdown, Scale bar, 100 μm. **d**-**f** Nude mice were injected subcutaneously with A549 tumor cells. After 7 days, tumor-bearing mice were treated with PBS (Veh), CEA (*i.p.* 50 μg/kg), SR18292 (45 mg/kg), H89 (10 mg/kg) or combined treatment every 3 days. Tumor growth was monitored every 3 days (**d**), tumor xenografts (**e**) and tumor volume (**f**) were assessed; *n* = 5. **g** Representative immunohistochemical images of PCNA, P-PKA and PGC-1α expression in A549 tumor tissues of indicated group were shown. Data are expressed as means ± SD. *P < 0.05, **P < 0.01, ***P < 0.001, by two-sided unpaired student’s *t* test

To investigate the oncogenic role of PGC-1α in NSCLC in vivo, PGC-1α-deficient A549 cells and control cells were subcutaneously injected into nude mice. The PGC-1α-deficient A549 cells displayed significantly smaller size compared to the control group (Fig. 6a). Moreover, tumor volume and weight were notably reduced following PGC-1α knockdown in comparison to the control group (Fig. 6b). Additionally, PGC-1α knockdown tumors exhibited lower levels of PCNA, P-PKA, and PGC-1α signals relative to the control tumor (Fig. 6c). Treatment with PGC-1α inhibitor SR18292 and PKA inhibitor H89 was found to inhibit the growth of A549 tumors in vivo (Fig. 6d-f). Furthermore, tumors treated with SR18292 and H89 displayed decreased levels of PCNA, P-PKA, and PGC-1α signals (Fig. 6g). These findings collectively indicate that CEA promotes NSCLC growth by modulating the PKA-PGC-1α axis.

## Discussion

CEA, a glycoprotein primarily used as a tumor marker, is of significant interest in understanding its function and relevance to malignant transformation, especially in colorectal cancer [5]. This study demonstrates that CEA plays a critical role in promoting the growth and spread of NSCLC both in vivo and in vitro. Specifically, CEA is shown to enhance the metabolism of fatty acids in lung cancer cells, leading to increased cell proliferation. Through RNA sequencing analysis, it was discovered that CEA alters lipid metabolism in NSCLC through the PKA-PGC-1α signaling pathways. Inhibition of either PKA or PGC-1α resulted in decreased growth of NSCLC tumor cells. These findings shed light on a new function of CEA in the progression of NSCLC.

The tumor microenvironment poses various stresses on cancer cells, such as nutrient and oxygen deprivation, along with extracellular acidification [21]. To progress, cancer cells must adapt and develop survival mechanisms in this challenging environment, often requiring metabolic reprogramming [22, 23]. This reprogramming enhances the ability of tumor cells to survive. Cancer cells exhibit increased de novo adipogenesis due to elevated expression of lipogenic enzymes like ATP citrate lyase (ACLY) and fatty acid synthase (FASN) [24]. Our study found that CEA-induced proliferation and metastasis of NSCLC cells depended on fatty acid metabolism. Treatment with CEA led to an increase in mitochondria and lipid droplet accumulation in A549 and H1299 cells. PGC-1α, a transcription coactivator crucial for regulating mitochondrial biogenesis and cellular energy metabolism [18], was significantly upregulated in CEA-treated NSCLC cells in our study. This upregulation was essential for NSCLC fatty acid metabolism and proliferation. Knockdown of PGC-1α reduced CEA-mediated NSCLC cell proliferation, indicating that CEA may regulate cell proliferation and lipid metabolism in NSCLC cells through the PGC-1α signaling pathway.

Recent studies have highlighted the significant roles of PGC-1α in various tumors, particularly its impact on tumor metabolism [25]. Targeting PGC-1α for its anti-tumor potential due to its influence on mitochondrial function has been explored [26–28]. Inhibitors such as SR18292 have demonstrated the disruption of metabolic processes, leading to energy depletion and oxidative damage to OXPHOS, ultimately impeding the proliferation and survival of tumor cells [29]. Our research aligns with these findings, showing that blocking PGC-1α signaling can inhibit CEA-induced proliferation and metastasis in non-small cell lung cancer cells. Despite its anti-tumor effects, PGC-1α may also play a role in suppressing tumor development under specific circumstances. Activation of PGC-1α has been associated with crucial aspects of CD8^+^ T cell function, including proliferation, mitochondrial biogenesis, and energy metabolism, potentially enhancing the efficacy of PD-1 combination therapy [30, 31]. Therefore, it is crucial to consider strategies that enhance the longevity and function of killer T cells when targeting PGC-1α in conjunction with anti-tumor immunotherapy. A deeper understanding of the pivotal role of PGC-1α in the tumor microenvironment offers promise for innovative approaches to treating non-small cell lung cancer.

CEA, a glycophosphatidylinositol-anchored protein lacking a cytoplasmic domain, requires a transmembrane interaction partner for intracellular signal transduction [5]. Previous research has demonstrated the co-localization of CEA and integrins in lipid rafts, influencing integrin-dependent signaling pathways such as phosphoinositide 3-kinase (PI3K) and AKT, both of which are integrin-linked kinases [18, 19]. Additionally, studies have indicated that cAMP can selectively enhance CEA expression [20]. Notably, our investigation revealed an increase in cAMP levels in lung cancer cells following CEA treatment. This study sheds light on how tumor cell-derived CEA promotes the growth and metastasis of NSCLC by activating the PKA and PGC-1α signaling pathways.

However, the limitations of our study should be acknowledged. Firstly, while CEA is associated with DDP resistance in NSCLC, the specific mechanisms of this relationship are still unclear. Further research is needed to understand how CEA influences DDP resistance in lung cancer cells for the development of effective clinical treatments. Secondly, although CEA-induced NSCLC proliferation is connected to the PKA-PGC-1α signaling pathway, it is possible that other fatty acid metabolism pathways also play a role in promoting NSCLC growth and metastasis. Lastly, the potential impact of CEA on anti-tumor immunity cannot be overlooked, as it may serve as an immunosuppressive factor in the tumor microenvironment, potentially contributing to NSCLC progression.

In summary, our study demonstrates that CEA triggers proliferation and migration in NSCLC, upregulates genes related to fatty acid metabolism and increases PGC-1α expression. This process is dependent on the PKA-PGC-1α pathway for NSCLC proliferation. Inhibition of PKA or PGC-1α leads to decreased fatty acid metabolism and inhibition of NSCLC growth and metastasis. These findings suggest a regulatory function of CEA in NSCLC cell proliferation and fatty acid metabolism.

## Materials and methods

### Cell lines and treatment

Human lung carcinoma A549 and H1299 cells were purchased from Type Culture Collection of the Chinese Academy of Sciences (Shanghai, China) in 2015. All cell lines were examined Mycoplasma-free using MycAwayTM-Color One-Step Mycoplasma Detection Kit (Yeasen Bio-technol) and these cells were authenticated and certified by ChengDu Nuohe Biotech co., LTD (Sichuan, China). Cells were cultured in DMEM medium containing high glucose supplemented with 10% FBS and 100 U/mL penicillin/streptomycin.

### Animals and tumor models

Nude mice were purchased from the Animal Institute of the Academy of Medical Science (Beijing, China). These mice were kept in specific pathogen-free conditions under a 12 h light cycle, and were given a regular chow diet at Chongqing University Cancer Hospital. A549 cells (1 × 10^6^) were implanted subcutaneously into male nude mice [32, 33]. Tumor growth was measured using calipers every 3 days. Tumor volume was calculated as follows: V = (length × width^2^) × 0.5. For SR18292 (SR, 45 mg/kg, Cat No. HY-101491, MCE) and H89 (10 mg/kg, Cat No. HY-15979A, MCE) treatment studies, treatment was given intraperitoneally every 3 days once tumors approximately 100 mm^3^ until the mice were sacrificed [34]. All animal experiments were approved by the ethics Committee of the Chongqing University Cancer Hospital, Chongqing, China. All animal studies were conducted in accordance with the national and international Guidelines for the Care and Use of Laboratory, and with the approval of the Animal Care and Use Committee (IACUC) of Chongqing University Cancer Hospital and complied with the Declaration of Helsinski.

### Flow Cytometry (FCM)

The A549 and H1299 cells were prepared and blocked with rat IgG (10 μg/mL; Sigma) for at least 20 min on ice. After that, they were washed with staining buffer (PBS containing 2% FBS). Then, the cells were labeled with the indicated antibodies (1:100) for 30 min at 4 °C. Dead cells were excluded using a Fixable Viability Dye Efluor 780 (1:1000; Cat No. 65–0865-14, eBioscience). Bodipy558/568 (Cat No. D3835) was obtained from Thermo Fisher Scientific. Mito-Tracker Green probes (Cat No. C1048) were from Beyotime (Shanghai, China). Flow cytometry (FCM) was performed on BD FACS Canto II platforms, and the results were analyzed with FlowJo software version 10.0.7 (TreeStar). Sorting was performed on a BD FACSAria II instrument (BD Biosciences).

### Colony formation assay

The A549 and H1299 cells were adjusted to 1 × 10^3^ cells/mL. Cell suspension was supplemented with culture medium and seeded in the 6-well plate. Then, these cells were uniformly dispersed and cultured for 2–3 weeks. Cell growth was terminated and the culture medium was discarded. The cells were treated by methanol for 15 min and dyed with crystal violet staining solution for 10 min. The colony number visible to naked eyes was counted, and the colony rate = (colony number/seeded cell number) × 100%.

### CCK-8 assay

The A549 and H1299 single cell suspension was diluted and adjusted to 1 × 10^4^ cells/mL. 200 μL of the diluted cell suspension was absorbed and seeded in the 96-well plate. For FFAs treatment, A549 cells were treated with 0.4 mM FFAs (mixture solution of palmitate acid (PA) and oleic acid (OA) as exogenous FFAs) for indicated times [16]. The experiment was completed by following the instructions of CCK-8 kit (Dojindo, Tokyo, Japan).

### Apoptosis assays

The A549 cells were treated with different concentrations of CEA, DDP (Cat No. ST1164, Beyotime) or combined for 24 h. Apoptotic ratio was measured by Annexin V-FITC/PI double staining kit (Cat No. FXP018, Beijing 4A Biotech) according to the manufacturer’s protocol. Caspase-3 activity was measured by kit (Cat No.C1116, Beyotime) according to the manufacturer’s protocol.

### Seahorse XFp metabolic assays

A549 cells were seeded at 1 × 10^4^ cells/well in 96-well plates for 3–4 h to allow adherence to the plate. Then, they were treated with CEA (50 ng/mL) for 24 h. OCR were measured as previously described [35].

### Transfection of shRNAs in A549 cells

Three pLKD-CMV-EGFP-2A lentivectors containing shRNAs targeting *PPARGC1A* or scrambled shRNA lentivector were obtained from Genechem (Shanghai, China). A549 cells were plated at 1 × 10^6^ cells per ml in 6-well plates and transduced with lentiviral particles (at MOI of 20) with 5 µg/ml Polybrene (GenePharma). Cells were harvested and screened for further experiments 3 days after transfection.

### ELISA

Intracellular cAMP expression in A549 cells were measured using a cAMP ELISA kit (Cat No. ab138880, Abcam) according to the manufacturer’s instruction.

### Immunohistochemistry

Formalin-fixed paraffin-embedded tumor samples slides were incubated in 0.3% H_2_O_2_ in methanol for 30 min to block endogenous peroxidase activity. Antigens were retrieved with 10 mmol L-1 sodium citrate (pH 6) for 5 min in a pressure cooker. The slides were then incubated with the antibodies (anti-PCNA (1:100, Cat. No. 2586S, Cell Signaling Technology), P-PKA (1:100, Cat. No. 9621S, Cell Signaling Technology), PGC-1α (1:100, Cat. No. 66369–1-Ig, Proteintech) antibody (1:100, Cat. No. 13684S, Cell Signaling Technology) at 4℃ overnight. The slides without treatment with primary antibody served as negative controls. Then, the sections were imaged on a phase contrast microscopy (Leica, Wetzlar, Germany).

### Quantitative real-time PCR

Total RNA was extracted using RNAiso Plus (TAKARA, Japan) and the RNA concentration was measured using NanoDrop 2000 (Thermo Scientific). 1 µg total RNA was converted to complementary DNA (cDNA) using the PrimeScript RT-PCR Kit (RR014A, Takara) according to the manufacturers’ instructions. qPCR was performed using TB Green Fast qPCR Mix Kit (RR430A, Takara) on a CFX384 system (BIO-RAD), and the relative quantification (2^−ΔΔCt^) method was used to analyze gene expression. β-actin mRNA was used as a reference for mRNA quantification. All qPCR experiments were repeated at least three times.

### RNA sequencing library construction

Total RNA was extracted from A549 cells after CEA or vehicle treatment. The RNA-seq library for these RNA samples was constructed according to the strand-specific RNA sequencing library preparation protocol. mRNA transcripts were enriched by two rounds of poly-(A +) selection with Dynabeads oligo-(dT) 25 (Invitrogen) before library construction. The prepared libraries were sequenced on an Illumina Novaseq 6000 platform.

### Western blotting

Cells were lysed by RIPA lysis buffer, and the cell lysates were incubated on ice for 30 min and centrifuged at 13,000 g, 4 °C for 15 min before the supernatant was collected. Western blot analysis was performed as previously described [36]. The primary antibodies included anti-PGC1-α (1:1,000; Cat No. 2178S, Cell Signaling Technology), anti-caspase-3 (1:1,000; Cat No. 66470-2-Ig, Proteintech), anti-p-PKA (1:1,000; Cat No. 4781S, Cell Signaling Technology).

### Statistical analysis

Statistical parameters and *n* values are indicated in figure legends. All results were confirmed in at least three independent experiments and were expressed as means ± SD. Student’s *t*-test and one- or two-way ANOVA were used for calculation statistical significance with GraphPad Prism software (version 8.0). For survival curves, the Kaplan–Meier method was used, and the differences in survival curves were analyzed using the log-rank test. *P* < 0.05 was considered statistically significant.

### Supplementary Information

**Supplementary file 1.**

## Data Availability

The experimental data sets generated and/or analyzed during the current study are available from the corresponding author upon reasonable request.
