# Supplementary material for: Carcinoembryonic antigen potentiates non-small cell lung cancer progression via PKA-PGC-1ɑ axis
Source: Mol Biomed. 2024 May 24;5:19. doi: 10.1186/s43556-024-00181-3 (PMC11116303; doi:10.1186/s43556-024-00181-3)
Supplement: Supplementary file 1 [file 43556_2024_181_MOESM1_ESM.doc]

Supplementary Materials for

**Carcinoembryonic antigen potentiates non-small cell lung cancer progression *via*** **PKA-PGC-1ɑ axis**

Juan Lei^1^, Lei Wu^1,2^, Nan Zhang^2^, Xudong Liu^2^, Jiangang Zhang^1^, Liwen Kuang^2^, Jiongming Chen^2^, Yijiao Chen^2^, Dairong Li^1,*^, Yongsheng Li^1,2,*^

^1^Department of Medical Oncology, Chongqing University Cancer Hospital, Chongqing 400030, China

^2^School of Medicine, Chongqing University, Chongqing 400030, China

^*^Correspondence: Dairong Li (lidairong@sohu.com); Yongsheng Li ([lys@cqu.edu.cn](mailto:lys@cqu.edu.cn)). Department of Medical Oncology, Chongqing University Cancer Hospital, Chongqing 400030, China

**Declaration of Competing Interest:** No potential conflicts of interest were disclosed.

**Keywords:** CEA, non-small cell lung cancer, fatty acid metabolism, PGC-1α, anti-tumor therapy

**Funding:** This work was supported by the Major International (Regional) Joint Research Program of the National Natural Science Foundation of China (No. 81920108027), Natural Science Foundation of Chongqing (cstc2020jcyj-msxmX0516), and Funding for Chongqing Young and Middle-Aged Medical Excellence Team.

**Supplementary Figures**


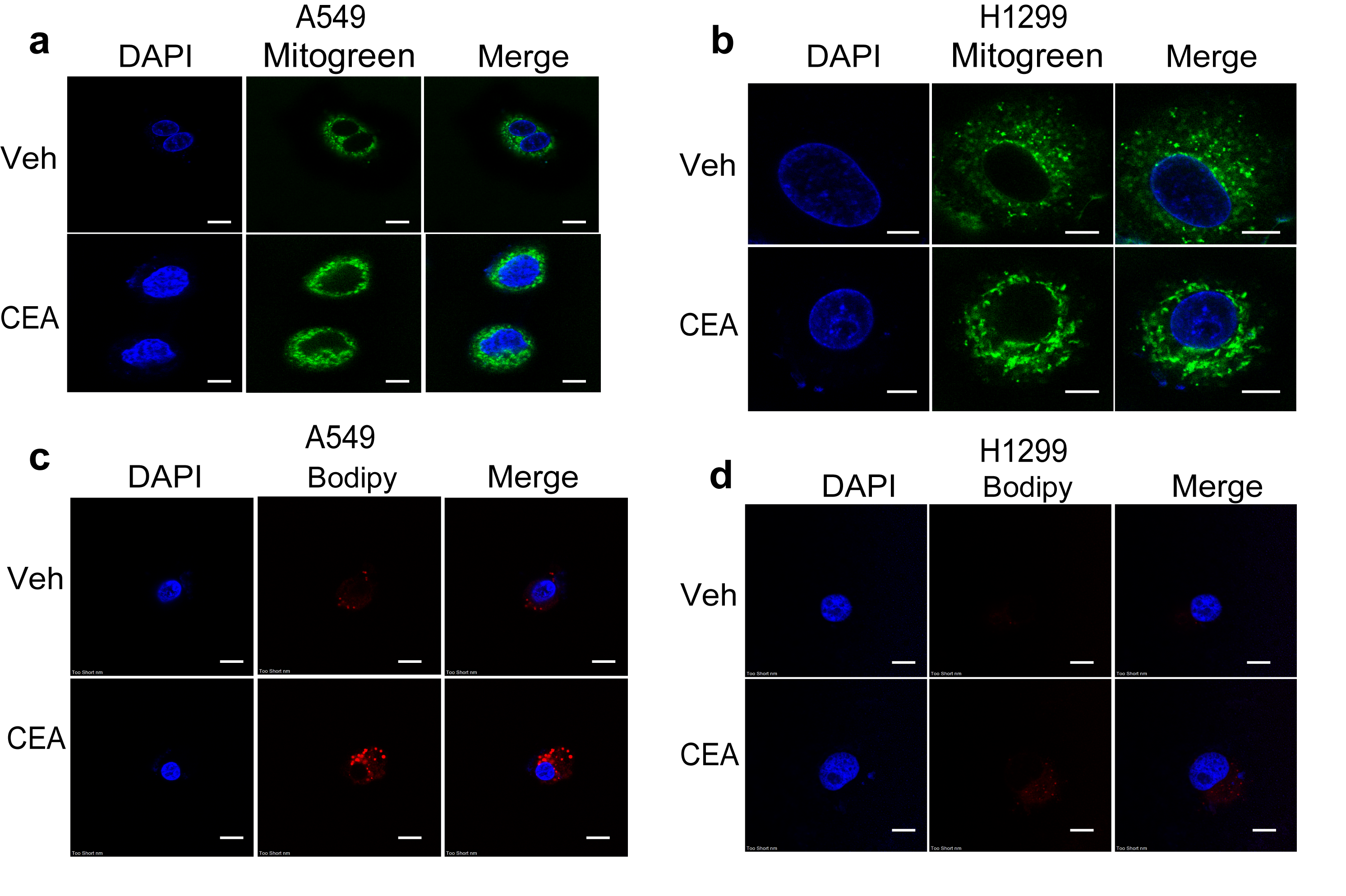
**Supplementary Fig. 1. Representative images of mitogreen and Bodipy staining of A549 and H1299 cells. a, b** A549 (**a**) and H1299 (**b**) cells were treated with CEA (50 ng/mL) for 24 h, Representative images of mitogreen staining. **c, d** Representative images of Bodipy 558/568 staining of A549 (**c**) and H1299 (**d**) cells after CEA (50 ng/mL) treatment for 24 h.


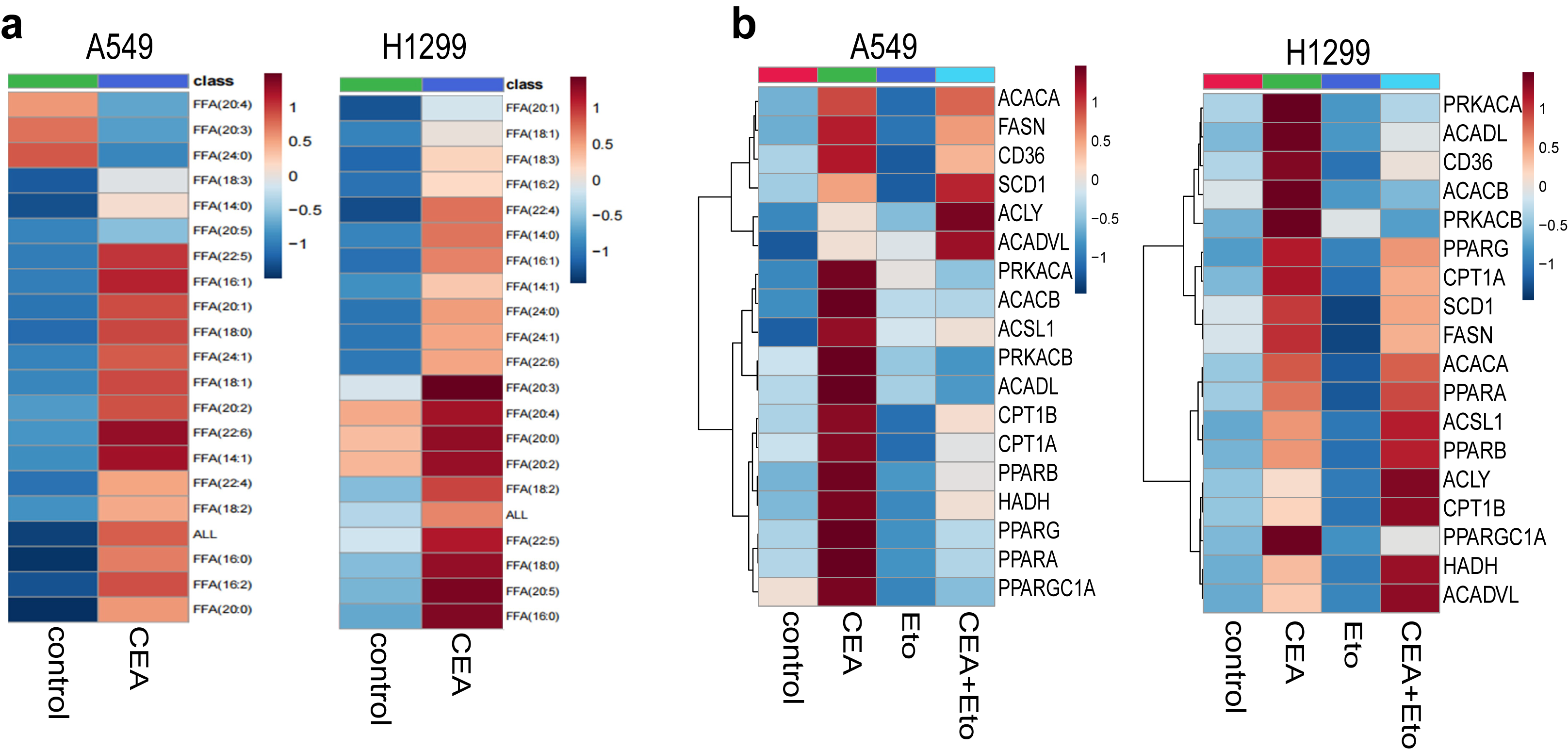


**Supplementary Fig. 2. CEA regulates fatty acid metabolism in A549 and H1299 cells. a** A549 and H1299 cells were treated with CEA (50 ng/mL) for 24 h, fatty acid intermediates in A549 and H1299 cells were determined by liquid chromatography-mass spectrometry (LC-MS). **b** A549 cells were treated with CEA, Eto (200 µM) or combined for 24h, then fatty acid metabolism related genes were determined.


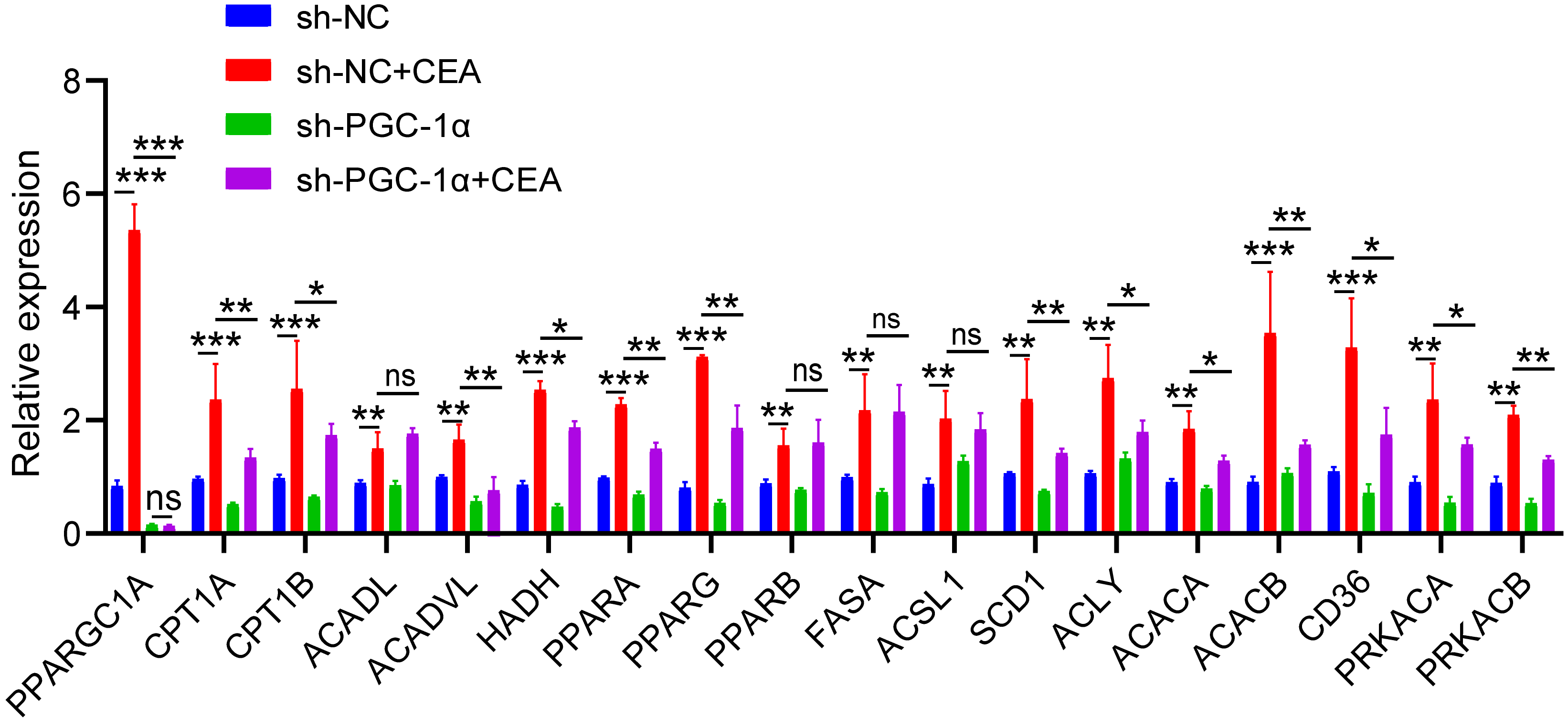
**Supplementary Fig. 3. CEA regulates fatty acid metabolism through PGC-1α in A549 cells.** A549 cells after *PPARGC1A* was knocked down and CEA treatment for 24 h, fatty acid metabolism related genes were determined. *n =*3. Data are expressed as means ± SD. **P <* 0.05, ***P* < 0.01, ****P <* 0.001, by two-sided unpaired student’s *t* test.


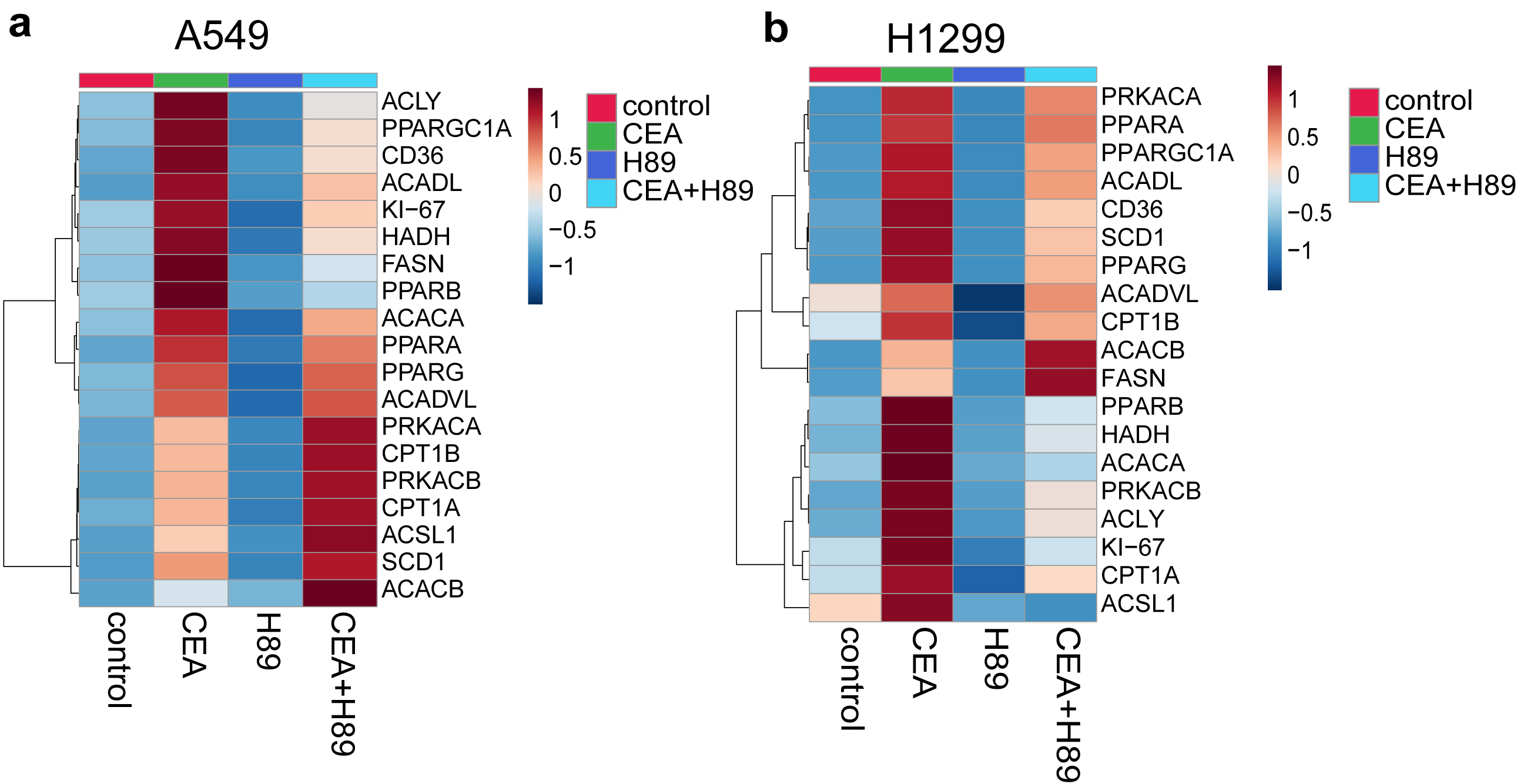


**Supplementary Fig. 4. CEA activates PGC-1α and fatty acid metabolism *via* PKA signal activation. a, b** A549 (**a**) and H1299 (**b**) were treated with H89 in the presence or absence of CEA (50 ng/mL) for 24 h, fatty acid metabolism related genes expression was determined by qPCR.
